# A Bilingual Arabic-English Ambient AI Scribe for Clinical Documentation: Prospective Evaluation Study

**DOI:** 10.2196/83335

**Published:** 2026-03-24

**Authors:** Umair Tahir Khan, Ammar Tahir Khan, Waleed Aljaadi, Razan Alhadlaq, Zahran Baqashmer, Yasin Alsafi, Yousef Alomran, Maha Al Rusaiyes, Muaddiyah Radif, Tahir Naeem Khan, Saleh Abdullah Saleh Altamimi

**Affiliations:** 1 Sahl AI, Anas Ibn Malik, Riyadh, 13521, Saudi Arabia; 2 King Saud Medical City, Riyadh, Saudi Arabia; 3 Riyadh First Health Cluster, Riyadh, Saudi Arabia; 4 IQVIA, Livingston, United Kingdom

**Keywords:** artificial intelligence, AI, natural language processing, speech recognition software, clinical documentation, physician burnout

## Abstract

**Background:**

Medical ambient artificial intelligence (AI) scribes reduce documentation burden, but the current evidence is almost entirely from English systems. In the Arabic-speaking world, physicians converse mainly in Arabic and write clinical notes in English, adding cognitive burden. Due to scarce corpora in the Arabic language, the development of Arabic-enabled AI speech technologies has been challenging. Here, we address this gap by developing and evaluating a bilingual Arabic-English medical AI scribe.

**Objective:**

This study aims to evaluate the feasibility and performance of a bilingual medical Arabic-English ambient AI scribe, Sahl AI, using a full end-to-end methodology from raw audio to clinical note.

**Methods:**

A prospective, single-arm feasibility pilot study was conducted in 2 stages across outpatient clinics, inpatient services, and primary care clinics within Riyadh First Health Cluster: development and implementation. In stage 1 (development), consultation audios were collected and manually annotated to fine-tune the AI pipeline. Version 1 of Sahl AI underwent feasibility evaluation using a modified 9-item Physician Documentation Quality Instrument (PDQI-9) framework based on Likert-scale ratings. Subsequently, in stage 2 (implementation), the AI pipeline was fine-tuned, and version 2 was evaluated as a real-world deployment in family medicine clinics. Independent reviewers evaluated clinical notes. Physician experience was captured using targeted surveys.

**Results:**

During stage 1, the AI pipeline was fine-tuned, producing version 1 of Sahl AI, which was tested in 64 clinical encounters for technical feasibility. This evaluation yielded a mean modified PQDI-9 score of 42.2 (SD 2.98) out of 45, with the model scoring 4.35 (SD 0.82) out of 5 in the accuracy domain. Following further optimization, version 2 of Sahl AI was evaluated during stage 2, using 55 real-world consultations assessed by 2 independent physician evaluators. Sahl AI achieved mean modified PDQI-9 scores of 42.4 (SD 1.84) out of 45 for Arabic and 37.8 (SD 1.10) out of 40 for English. On the Likert scale, for the accuracy domain, the mean score was 4.53 (SD 0.46) out of 5 for Arabic compared with 4.77 (SD 0.37) out of 5 for English (*P*=.06). Internal consistency (mean 4.94, SD 0.17) and comprehensibility (mean 4.89, SD 0.21) were the top-rated domains. The targeted survey of a larger cohort of 22 family medicine physicians using Sahl AI showed that most responding physicians agreed that notes were comprehensive and perceived potential time savings and reduced burnout.

**Conclusions:**

Sahl AI, a bilingual Arabic-English medical ambient AI scribe, generates accurate and high-quality notes, and in targeted postimplementation surveys, most physicians agreed that it produced comprehensive notes and perceived potential benefits for time savings and stress or burnout. This provides the first empirical basis for rigorous end-to-end ambient AI scribe evaluation in low-resource languages such as Arabic.

## Introduction

Physicians devote a significant portion of their time to the administrative task of documenting medical encounters with patients [1]. Clinical documentation serves critical functions, including care continuity, quality assurance, and in many jurisdictions, medico-legal protection. It is an essential component for ensuring patient safety and also impacts subsequent tasks, such as submitting insurance claims [2]. The digitization of medical records and the widespread adoption of electronic health record systems have substantially increased the documentation burden on physicians, leading to increased physician burnout [3]. Furthermore, clinical documentation has become a distraction, changing the traditional doctor-patient relationship into a doctor-computer-patient interaction [4].

Clinical documentation is typically completed either during or after patient encounters. Documenting in real time may interrupt the clinical interaction, whereas postvisit documentation may add to physician workload and relies on recall rather than contemporaneous capture. Ambient artificial intelligence (AI) scribes automate clinical note generation and improve the doctor-patient relationship, freeing physicians to focus on personalized care [5,6]. This study evaluates Sahl AI, an AI tool for Arabic and English, using the modified 9-item Physician Documentation Quality Instrument (PDQI-9) tool. This research is unique in its dual-language focus and is particularly relevant in Arabic-speaking countries where clinical encounters are conducted in Arabic, while medical documentation is recorded in English. This supports health care providers who routinely switch between languages during consultations when eliciting patient histories and documenting care.

Arabic is often categorized as a low-resource language in speech technologies due to its complex characteristics. These include its writing system with positional variance and diacritical marks, phonology that features emphatic consonants and vowel length distinctions, and grammar that involves root-based morphology and case-gender agreement. The variability is further compounded by numerous dialects and diglossia, as well as influence from other languages and its significant religious context. This necessitates ongoing translation by the physicians, introducing cognitive strain. Although ambient AI scribes offer a potential solution to mitigate this burden, the effectiveness of bilingual systems in such linguistically complex environments remains insufficiently examined. Therefore, the aim of this study was to develop and assess the feasibility and physician experience of a bilingual Arabic-English medical ambient AI scribe in routine clinical practice.

## Methods

### Study Design and Objectives

The study was a prospective, single-arm feasibility pilot study evaluating the performance of Sahl AI, a bilingual ambient AI scribe, in routine physician-patient consultations. The primary objectives were to (1) fine-tune an AI pipeline for automated clinical documentation generation and subsequently (2) evaluate the usability of Sahl AI and its impact on the physicians’ workflow and the quality of documentation.

### Study Setting and Duration

**Figure 1.**
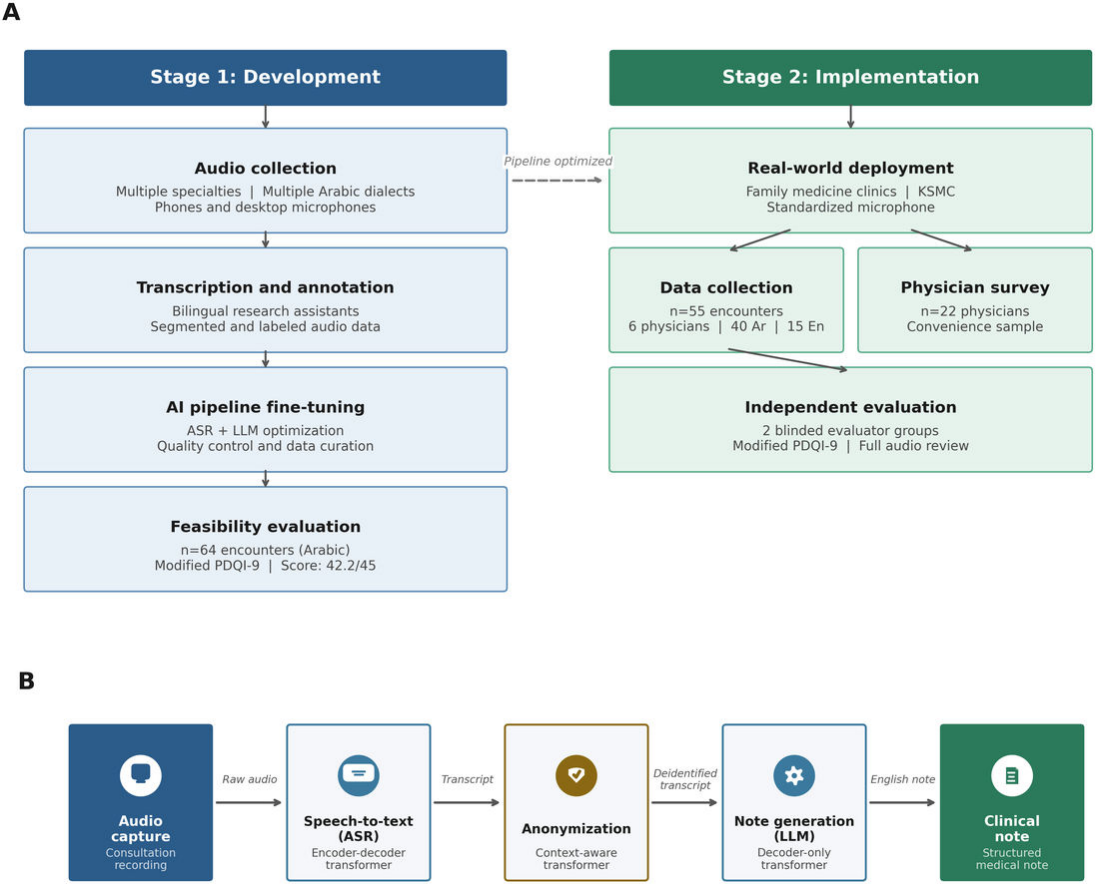
(A) Two-stage study design. Stage 1: iterative development including multidialect Arabic audio collection, manual transcription by bilingual research assistants, ASR and LLM fine-tuning, and feasibility evaluation (n=64 encounters; mean 9-item Physician Documentation Quality Instrument [PDQI-9] score: 42.2/45, SD 2.98). Stage 2: real-world implementation at family medicine clinics (King Saud Medical City [KSMC]), with prospective data collection (n=55 encounters; 40 Arabic, 15 English), independent blinded evaluation using the modified PDQI-9, and a parallel physician experience survey (n=22). Dashed arrow indicates pipeline transfer from development to implementation. (B) Artificial intelligence (AI) pipeline architecture: audio capture, encoder-decoder ASR, anonymization via context-aware transformer for personally identifiable information (PII) deidentification, and decoder-only LLM for clinical note generation. ASR: automatic speech recognition; LLM: large language model.

The study was conducted between December 23, 2023, and November 28, 2024, across outpatient clinics, inpatient services, and primary care centers within Riyadh First Health Cluster (Riyadh, Saudi Arabia). The study comprised 2 stages: (1) a development stage, during which data collection and a feasibility evaluation were performed, and (2) an implementation stage, in which the fine-tuned AI pipeline was assessed in a real-world clinical setting (Figure 1A).

During stage 1, participating specialties included, but were not limited to, general and internal medicine, general surgery, pediatrics, family medicine, obstetrics and gynecology, cardiology, and respiratory medicine. Both new and follow-up patient encounters were included, provided eligibility criteria were met and physicians were able to conveniently record audio conversations during clinical encounters. During stage 2, new patient encounters were sampled during designated family medicine clinic sessions when participating physicians were available. Verbal consent was obtained from patients by the treating physicians and was documented in the clinical notes.

### Eligibility Criteria

The inclusion criteria were as follows: patients of all ages and sexes who provided informed consent for the use of Sahl AI during their clinical consultation. For pediatric patients, consent was obtained from a parent or legal guardian.

The exclusion criteria were as follows: patients with cognitive impairment who were unable to provide informed consent.

### Collection of Audio Data for Fine-Tuning

During stage 1, audio data were collected to optimize and fine-tune the AI pipeline using Sahl AI’s proprietary Data Collection Pipeline. Audio streams from physician-patient conversations were segmented into shorter audio chunks to facilitate accurate labeling. Participating physicians received training on audio recording using various devices and on obtaining informed consent from patients. The recorded audio was securely stored on cloud servers in compliance with the Kingdom of Saudi Arabia’s Personal Data Protection Law (PDPL). The audio data were initially retained for 6 months, with the retention period later extended to 1 year following approval of an ethics amendment.

Recordings were collected across multiple clinical specialties during stage 1 using physicians’ personal mobile phones as well as desktop microphones, including Anker PowerConf S360 USB Speakerphones and Kaysuda SP300 Bluetooth Speakerphones. A team of bilingual research assistants fluent in Arabic and English reviewed and edited the transcribed audio segments to ensure high-quality labeled datasets for AI pipeline optimization. The dataset encompassed a wide range of Arabic dialects, including those spoken in Saudi Arabia, Yemen, Egypt, Sudan, the Levant, Kuwait, and North Africa, as well as simplified Arabic spoken by nonnative speakers and diverse English accents. Data scientists subsequently curated the labeled data, excluding poor-quality audio segments identified during review by the research assistants.

The AI pipeline was iteratively tested with internal evaluation metrics before being piloted with physicians during stages 1 and 2 of the study. The pipeline (Figure 1B) comprised multiple components requiring optimization for clinical deployment. In particular, the speech-to-text engine was based on a transformer-based encoder-decoder automatic speech recognition architecture, in which the encoder transforms raw audio signals into high-level semantic representations and the decoder generates the corresponding text transcription. Prior to clinical note generation, transcripts were processed through a deidentification layer that used a context-aware transformer model to detect and redact personally identifiable information. The large language model component was a decoder-only autoregressive transformer trained using causal language modeling.

Fine-tuning focused on improving robustness to spontaneous clinical dialogue, variability in dialects, and background noise commonly encountered in outpatient and inpatient environments. Model training prioritized transcription accuracy for medical conversations. To minimize overfitting and prevent information leakage, training and evaluation datasets were strictly separated.

### Feasibility Study

During stage 1, following the fine-tuning and optimization of the AI pipeline, version 1 of Sahl AI was evaluated in a controlled setting to assess feasibility outside of routine clinical practice. This evaluation involved role-plays and mock conversations between physicians, as well as prerecorded simulated physician-patient conversations generated by nonphysicians. These audio recordings were processed by the AI pipeline to generate clinical notes, enabling the assessment of the ambient AI scribe’s performance in the Arabic language. Collectively, these activities constituted the feasibility component of the development stage.

Clinical notes were generated in accordance with the documentation requirements of the Central Board for Accreditation of Healthcare Institutions and included standardized headings, such as chief complaint, history of present illness, past medical or surgical history, allergy, medication, social history, family history, physical examination findings, diagnosis, and plan. Participating physicians evaluated the AI-generated clinical notes after completing the role-play scenarios or listening to the prerecorded simulated conversations.

In total, 64 independent assessments were conducted using a modified PDQI-9 (Table 1), a tool that has been previously validated and used in recent evaluations of ambient AI scribes [7,8]. For the purposes of this study, the PDQI-9 tool was adapted to reflect Arabic-language and bilingual audio-to-text workflows. Specifically, the original domains of “up-to-date” and “synthesized” were replaced with “free from hallucination” and “translated accurately to English,” respectively. These modified domains have not been formally validated and were used for exploratory assessment only.

**Table 1.** Nine-item Physician Documentation Quality Instrument (PDQI-9) tool measuring the quality of the clinical note across 9 dimensions^a^.

Domain	Description
Accurate	The note is true. It is free of incorrect information. For example, if the history is about chest pain and the note mentions abdominal pain, then this represents that the note was not accurate.
Thorough	The note is complete and free from omission and documents all of the issues of importance to the patient. For example, if a symptom about visual loss was discussed and was important to the note but not mentioned, then this represents that the note was not thorough.
Useful	The note is extremely relevant, providing valuable information and/or analysis.
Organized	The note is well formed and structured as per CBAHI^b^ recommendations in a way that helps the reader understand the patient’s clinical course.
Comprehensible	The note is clear, without ambiguity or sections that are difficult to understand.
Succinct	The note is brief, to the point, and without redundancy.
Internally consistent	No part of the note ignores or contradicts any other part. For example, if 1 section of the note mentions that the patient has asthma but another section says no history of asthma, then this is not internally consistent.
Free from hallucination	The note is free of hallucination and only contains information verifiable by the audio conversation. For example, if the note mentions that the patient smokes and drinks alcohol when it was not at all discussed in the note, then this represents hallucination.
Translated accurately to English	The note reflects the Arabic conversation between the physician and the patient accurately in the English language.

a Each domain was scored from 1 to 5 (Likert scale), with 1 being “not at all” and 5 being “extremely.”

b CBAHI: Central Board for Accreditation of Healthcare Institutions.

### Real-World Testing

Based on the findings from stage 1, the AI pipeline was further optimized to improve performance prior to real-world deployment. During stage 2, the model was deployed in family medicine clinics for real-world testing involving both new and follow-up patient encounters. From a larger cohort of physicians, 6 physicians who consented to participate received at least 30 minutes of training on the effective use of the software prior to study initiation.

Physician-patient conversations and the corresponding unedited clinical notes generated by Sahl AI were recorded and subsequently evaluated by 2 independent evaluators. Evaluators listened to the complete audio recordings and assessed the associated unedited clinical notes using the modified PDQI-9 tool. To reduce the likelihood of identifying the physician speaker, evaluators were recruited from clinical specialties different from those of the participating physicians; however, complete anonymization could not be guaranteed, as the evaluators were recruited from the same health cluster. Evaluators were blinded to the identities of both patients and physicians. However, as the raw audio was not modified to redact personally identifiable information, complete blinding cannot be assured. Scores from evaluators were averaged prior to calculating the final modified PDQI-9 domain scores.

The evaluation process involved 2 evaluator groups. Evaluator group 1 comprised a single reviewer, while evaluator group 2 consisted of 2 reviewers who divided the assigned clinical notes between them. Each clinical note was reviewed by 2 evaluators—1 from each group. In cases where domain-level scores differed by 2 or more points, both evaluators were asked to rereview the clinical notes and reconcile discrepancies.

To ensure consistency across recordings, a standardized microphone (Kaysuda SP300 Bluetooth Speakerphones) connected to a laptop computer was used for all consultations. Audio recordings were performed by trained research assistants and uploaded directly through the Sahl AI software. Following each consultation, Sahl AI generated a single clinical note immediately using the recorded audio, which was then reviewed in its unedited form by the independent evaluators.

Clinical encounters were categorized as “Arabic” or “English” based on the predominant language of the consultation, as initially determined by research assistants and subsequently confirmed by the evaluators. Code-switched encounters involving substantial use of both languages were classified according to the language comprising the majority (>50%) of clinical content. Regardless of input language, Sahl AI generated all clinical documentation exclusively in English.

### Physician Experience

Following real-world deployment in the family medicine department, a targeted physician experience survey was conducted to capture feedback from clinicians with the direct hands-on use of Sahl AI. The survey was completed between 29 July and 28 November 2024 by 22 physicians. Eligibility was limited to physicians who had used the system in at least 3 clinical encounters, ensuring sufficient exposure to evaluate its impact on clinical workflow. Survey items evaluated perceived note comprehensiveness, time savings, ease of use, impact on physician burnout, and impact on clinical workflow using a 5-point Likert scale ranging from strongly agree to strongly disagree. The survey was designed as a descriptive assessment of user experience among active users rather than a population-level evaluation.

### Statistical Analysis

During the development stage, individual modified PDQI-9 scores were recorded for each physician’s assessment. In contrast, during the implementation stage, the modified PDQI-9 scores were calculated by calculating the mean scores from 2 independent evaluators for each domain and then summing these domain-level means. The overall modified PDQI-9 scores were reported as mean (SD) and were calculated out of a maximum total score of 45 for Arabic conversations and 40 for English conversations, as the “translated accurately to English” domain was not applicable to English consultations. Since the modified PDQI-9 domain scores were ordinal Likert-scale measures and group sizes were unbalanced (40 Arabic vs 15 English consultations), comparisons between Arabic and English groups were conducted using nonparametric Mann-Whitney *U* tests. Differences between evaluator scores were quantified using the mean absolute deviation (MAD), calculated as the average of the absolute differences between paired domain scores. MAD was computed both overall and at the domain level. Descriptive statistics, including means and frequencies, were used to summarize the responses from the physician experience questionnaire.

### Ethical Considerations

This study was approved by the institutional review board of King Saud Medical City (protocol H1R1-18-Sep23-01). Verbal informed consent was obtained from all participating patients prior to audio recording and was documented in the medical record. The use of verbal consent was approved by the institutional review board given the minimal-risk nature of the study and the practical limitations of obtaining written consent during routine clinical encounters without disrupting workflow. For pediatric patients, consent was obtained from parents or guardians. All data collection, storage, and analysis procedures were conducted in accordance with confidentiality requirements and complied with the Saudi Data and Artificial Intelligence Authority PDPL [9]. No patient demographic information was collected, and independent evaluators assessed clinical notes without access to any identifying data. Patients did not receive financial compensation for participation, and physicians participated as part of their routine clinical duties.

## Results

### Feasibility Pilot

During the development stage of the study, feasibility testing was conducted on 64 Arabic-language conversations, with the results summarized in Table 2. The overall mean modified PDQI-9 score across all domains was 42.2 (SD 2.98) out of 45. Clinical notes achieved the highest ratings in the domains of “useful,” “organized,” and “comprehensible,” with mean scores of 4.86 (SD 0.39), 4.87 (SD 0.34), and 4.85 (SD 0.35) out of 5, respectively. The accuracy domain of version 1 of the pipeline was rated 4.35 (SD 0.82) out of 5. Relatively lower scores were observed for the thorough (mean 4.42, SD 0.81) and free from hallucination (mean 4.45, SD 0.85) domains, highlighting areas for further improvement. The identified strengths and limitations informed the subsequent fine-tuning and optimization of the AI pipeline.

**Table 2.** Domain-level performance of Sahl AI-generated clinical notes during the development (n=64) and implementation (n=55) stages, assessed using the modified 9-item Physician Documentation Quality Instrument (PDQI-9) tool^a^.

Domain	Development stage^b^		Implementation stage^c^	
	Number of cases	Mean (SD)	Number of cases	Mean (SD)
Accurate	64	4.35 (0.82)	55	4.59 (0.45)
Thorough	64	4.42 (0.81)	55	4.55 (0.45)
Useful	64	4.86 (0.39)	55	4.74 (0.33)
Organized	64	4.87 (0.34)	55	4.54 (0.37)
Comprehensible	64	4.85 (0.35)	55	4.89 (0.21)
Succinct	64	4.84 (0.45)	55	4.49 (0.38)
Internally consistent	64	4.73 (0.60)	55	4.94 (0.17)
Free from hallucination	64	4.45 (0.85)	55	4.87 (0.22)
Translated accurately to English	64	4.79 (0.49)	40	4.85 (0.28)

a Values represent mean (SD) Likert-scale scores for each documentation quality domain. The overall mean modified PDQI-9 scores are reported separately for Arabic (out of 45) and English-translated (out of 40) conversations.

b Average for development stage: 42.2 (SD 2.98) out of 45.

c Average for implementation stage: Arabic: 42.4 (SD 1.84) out of 45; English: 37.8 (SD 1.10) out of 40.

### Real-World Implementation

During the implementation stage, real-world testing was conducted in family medicine clinics at King Saud Medical City (Riyadh First Health Cluster). Independent evaluators reviewed 55 new-patient consultations using the modified PDQI-9 tool; 40 consultations involved predominantly Arabic physician-patient conversations, and 15 involved English conversations. The mean modified PDQI-9 score was 42.4 (SD 1.84) out of 45 for Arabic consultations and 37.8 (SD 1.10) out of 40 for English consultations (Table 2).

Across all cases, clinical notes achieved the highest scores in the domains of “internally consistent,” “comprehensible,” and “free from hallucination” with mean scores of 4.94 (SD 0.17), 4.89 (SD 0.21), and 4.87 (SD 0.22) out of 5, respectively. The mean accuracy domain score for version 2 of the fine-tuned and optimized AI pipeline was 4.59 (SD 0.45) out of 5 across both language groups. Mean scores exceeded 4.5 out of 5 across all domains except for “succinct,” which scored 4.49 (SD 0.38), suggesting a tendency toward more verbose clinical notes.

**Figure 2.**
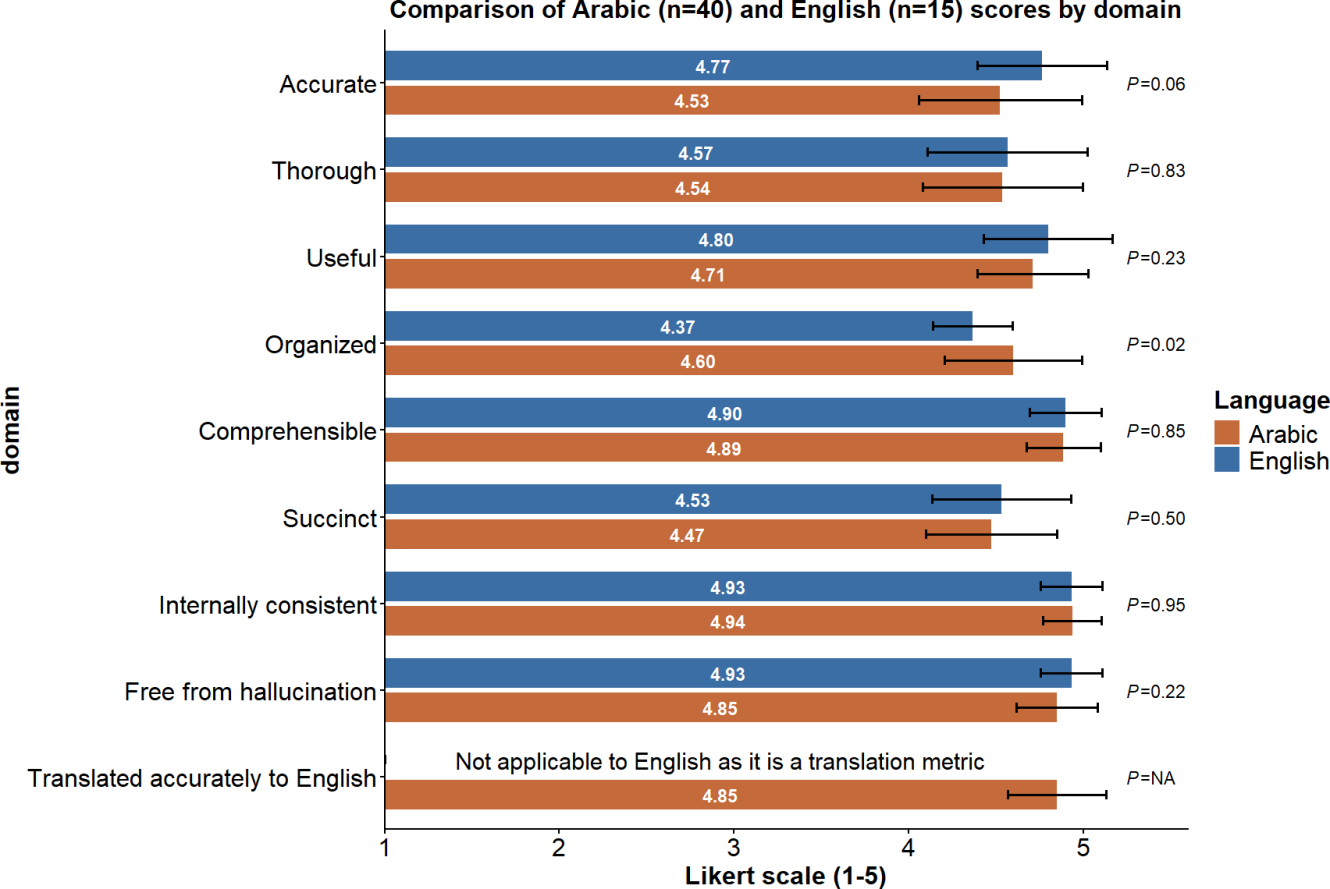
Comparison of mean Likert-scale scores for clinical notes generated by Sahl AI, as evaluated by independent reviewers, for Arabic and English conversations across each modified 9-item Physician Documentation Quality Instrument (PDQI-9) domain. Error bars indicate ±1 SD. Between-group differences were assessed using the Mann-Whitney *U* test. NA: not applicable.

Domain-level scores for Arabic and English encounters were analyzed separately and are presented in Figure 2. A statistically significant difference was observed in the “organized” domain, with higher scores for Arabic compared with English conversations (4.60, SD 0.39 vs 4.37, SD 0.23; *P=*.02). There was a trend to the English conversations having higher accuracy compared with Arabic conversations (4.77, SD 0.37 vs 4.53, SD 0.46; *P=.*06). No statistically significant differences were observed across the remaining domains.

### Interevaluator Agreement

Interevaluator agreement across modified PDQI-9 domains is presented in Table 3. Overall agreement between evaluator group 1 and evaluator group 2 was high, with a mean absolute deviation of MAD=0.36, indicating close concordance in scoring. Across individual domains, MAD values ranged from 0.13 to 0.66 on a 5-point Likert scale, demonstrating that evaluator ratings typically differed by <1 point. Exact agreement of scores ranged from 34.5% to 87.3%, while agreement within ±1 point on the Likert scale was consistently high across domains (96.4%‐100%).

**Table 3.** Interevaluator agreement between evaluator group 1 and evaluator group 2 across modified 9-item Physician Documentation Quality Instrument (PDQI-9) domains^a^.

Domain	Number of cases	MAD^b^	Exact agreement (%)	Agreement ±1 (%)	ICC^c^	Cohen kappa
Accurate	55	0.42	60	98.2	0.28	0.28
Thorough	55	0.40	60	100	0.35	0.35
Useful	55	0.35	65.6	100	0.13	0.13
Organized	55	0.60	43.6	96.4	0.12	0.12
Comprehensible	55	0.22	78.2	100	−0.07	−0.06
Succinct	55	0.66	34.5	100	−0.05	−0.05
Internally consistent	55	0.13	87.3	100	0.06	−0.05
Free from hallucination	55	0.26	74.5	100	0.00	0
Translated accurately to English	40	0.20	80	100	0.22	0.22

a Agreement is summarized using mean absolute difference, exact agreement (%), and agreement within +1 point on the Likert scale (%), intraclass correlation coefficient (2-way mixed effects, absolute agreement), and weighted Cohen kappa (squared weights).

b MAD: mean absolute deviation.

c ICC: intraclass correlation coefficient.

The highest interevaluator agreement was observed for the internally consistent (MAD=0.13; exact agreement 87.3%) and translated accurately to English domains (MAD=0.20; exact agreement 80%). Greater variability was observed in more subjective stylistic domains, including succinct (MAD=0.66; exact agreement 34.5%) and organized (MAD=0.60; exact agreement 43.6%). Despite lower exact agreement in these domains, agreement within ±1 point remained high (≥96.4%), supporting overall consistency between evaluators. To supplement the MAD analysis, the intraclass correlation coefficient (ICC; 2-way mixed effects, absolute agreement) and weighted Cohen kappa were calculated for each domain and are reported in Table 3. However, domain-level ICC and kappa values were markedly attenuated by ceiling effects, as 99% of the individual ratings were 4 or 5 on the Likert scale. Under such restricted variance, these metrics can yield paradoxically low or negative values despite high observed concordance; for example, the succinct and internally consistent domains each achieved 100% agreement within ±1 point yet produced negative ICC and kappa values. Given these distributional constraints, the MAD and percentage agreement metrics provide a more clinically meaningful assessment of interevaluator concordance in this setting.

### Physician Experience

Physician experience with Sahl AI was evaluated to assess its perceived impact on routine clinical practice. A larger pool of physicians who may not have contributed to the development or implementation stage but used Sahl AI in their clinical practice was surveyed. In total, 22 physicians responded; however, as the total number of invited physicians was not systematically recorded, a formal response rate cannot be calculated, and these results should be interpreted as descriptive feedback from a convenience sample of unknown proportion. The results are summarized in Table 4. Most responding physicians strongly agreed or agreed across all domains that Sahl AI produced comprehensive notes, produced more complete notes compared with current practice, had the potential to save time and reduce physician burnout, was faster, while having a positive impact on their clinical workflow.

**Table 4.** Summarization of the physicians’ responses to various statements related to the use of Sahl AI and impact on their clinical practice.

Question	Strongly agree, n (%)	Agree, n (%)	Neither agree nor disagree, n (%)	Disagree, n (%)	Strongly disagree, n (%)
Sahl AI produces comprehensive clinical summaries of the consultations	19 (86.4)	3 (13.6)	0 (0)	0 (0)	0 (0)
I feel that Sahl AI can produce more complete notes compared to current documentation practices	14 (63.6)	6 (27.3)	2 (9.1)	0 (0)	0 (0)
Sahl AI was faster and thus has the potential to save time in documenting comprehensive clinical summaries compared to the present practice	19 (86.4)	2 (9.1)	1 (4.5)	0 (0)	0 (0)
I feel that the use of the tool can help reduce physician stress/burnout	15 (68.2)	6 (27.3)	1 (4.5)	0 (0)	0 (0)
It was easy to use the Sahl AI tool	19 (86.4)	2 (9.1)	0 (0)	1 (4.5)	0 (0)
Sahl AI has the potential to positively affect my clinical workflow	16 (72.7)	4 (18.2)	2 (9.1)	0 (0)	0 (0)

## Discussion

### Principal Findings

This study demonstrates the feasibility and effectiveness of a bilingual Arabic-English medical ambient AI scribe in generating accurate, thorough, and clinically useful documentation in a real-world clinical setting. The study lays a foundation and benchmark for the successful deployment of medical AI technologies for low-resource languages, such as Arabic, and in the bilingual setting. The positive feedback from surveyed physicians regarding the tool’s ease of use, potential for time savings and reducing burnout, producing more complete notes and impact on clinical workflow provides preliminary, descriptive evidence of its value in clinical practice. The results are especially relevant to health care systems in Arabic-speaking settings, where improving documentation efficiency and quality remains a critical operational priority. Furthermore, to our knowledge, this study represents the first end-to-end evaluation of a medical ambient AI scribe spanning audio capture to finalized clinical note generation for the Arabic language, extending beyond prior work that has primarily focused on transcript-to-note pipelines.

### Comparison to Prior Work

There is a growing body of research evaluating the performance of ambient AI scribes in the English language, but their performance has not been rigorously assessed in other languages, particularly Arabic, which has multiple dialects and is considered a low-resource language in natural language processing due to a dearth of available datasets. This lack of evaluation poses a significant challenge, as the linguistic and cultural nuances of Arabic necessitate tailored solutions for the successful deployment of medical AI technologies in Arabic-speaking regions. Additionally, challenges in recording medical data, such as ensuring data privacy of health data and compliance with laws, such as PDPL, have contributed to this limitation. This study provides a crucial methodology for the collection of data and the training of ambient AI scribes in any language, especially low-resource languages, such as Arabic, which can help address these gaps and pave the way for the successful deployment of these technologies in diverse linguistic and cultural contexts.

Medical ambient AI scribes have been widely adopted in English-speaking health care settings and are generally reported to generate accurate and comprehensive clinical notes. For example, Tierney et al [7] reported a high documentation quality score of 48 out of 50 using a modified PDQI-9 when evaluating English physician-patient transcripts. In contrast, substantially lower scores have been reported in studies evaluating large language models, such as GPT-4, with PDQI-9 scores of 29.7 out of 45 in 1 study and 25 out of 35 using a modified PDQI-9 in another [10,11]. Kernberg et al [11] further noted that the AI-generated notes frequently omitted clinically important information, introduced hallucinated content, or contained factual inaccuracies and that note generation was not consistently reproducible. More recently, an English-language medical ambient AI scribe incorporating audio input in addition to transcripts was evaluated using a modified PDQI-9 tool, achieving a mean score of 41.9 out of 50 [12].

As a reference benchmark, the original PDQI-9 validation study categorized scores of 26.2 out of 45 as “terrible or bad,” compared with scores of 36.6 out of 45 as “good or excellent” [8]. Evidence in non-English and low-resource language settings remains limited. A recent study by Lee et al [13] reported a score of 50.2 out of 65 using a modified PDQI-9 tool for synthetic Arabic transcript-to-note generation. Additional efforts have been reported in other non-English contexts, including Spanish-language AI scribe implementations in surgical outpatient settings [14].

In this study, the total mean score was 42.4 (SD 1.84) out of 45 for Arabic conversations and 37.8 (SD 1.10) out of 40 for English conversations. As in prior studies, the assessment instrument was modified to suit the study context. As these modified domains have not been formally validated, the aggregate scores should be interpreted with caution and are not directly comparable to benchmarks established using the original PDQI-9 instrument. However, unlike previous research that primarily assessed note quality based on written transcripts, this study introduces a novel approach by evaluating notes generated directly from original audio recordings of physician-patient consultations. These audio-derived notes were subsequently reviewed by independent evaluators, offering a more authentic and context-rich basis for assessing note quality. This methodology improves existing studies by considering errors from speech-to-text engines, performing an end-to-end evaluation to better reflect real-world situations rather than considering transcripts as ground truth. By evaluating the entire process from audio input to textual output, the approach ensures that the findings are applicable to everyday use cases, enhancing the robustness and reliability of the results. Although the summary of prior work focuses on PDQI-9 and its derivatives for easier comparison, other studies have utilized Recall-Oriented Understudy for Gisting Evaluation (ROUGE), Bidirectional Encoder Representations from Transformers (BERTScore), Crosslingual Optimized Metric for Evaluation of Translation (COMET), and Sheffield Assessment Instrument for Letters (SAIL), among others, which have not been discussed here.

Surveyed physicians perceived that the tool improved note accuracy and quality compared to the clinical practice of typing notes, resulting in more comprehensive and complete documentation compared to current practice. Most responding physicians perceived that the use of Sahl AI can reduce cognitive overload and save time, resulting in a positive overall impact on their clinical workflow. Such findings have been confirmed by other studies, especially showing that the use of ambient AI scribes leads to time saved and reductions in cognitive burden for physicians [10,15-21]. However, a recent report by the Peterson Health Technology Institute concluded that while AI scribes may reduce clinician burnout, it is unclear whether it actually saves time and leads to financial gains [22]. The report concluded that differences in outcomes were likely due to organization-specific metrics, which had variation in how they were defined and measured. Nevertheless, the result of this study is in line with the growing consensus that AI scribes can enhance physician efficiency by alleviating the cognitive burden associated with comprehensive documentation.

### Limitations

This study used a single-arm design without a comparator, as comparative evaluation was outside the scope of this work. The absence of baseline comparisons in Arabic or bilingual settings with physician-authored documentation or generic large language models, both in the literature and in this study, limits the ability to draw conclusions regarding comparative effectiveness. Future studies incorporating head-to-head comparisons are required to establish relative performance.

The sample size was limited due to the practical challenges of collecting real-world audio data, which may affect the interpretability and generalizability of the findings. In addition, physicians used Sahl AI as a stand-alone web app that was not integrated into the electronic medical record. Electronic medical record integration is an important factor in simplifying clinical workflows and has been associated with the improved adoption of ambient AI scribes [21]. Real-world testing was conducted exclusively within the family medicine specialty; therefore, the findings may not generalize to other clinical specialties, and further evaluation across diverse clinical domains is warranted. Furthermore, the independent evaluation was conducted by physicians from the same health cluster; although evaluators were blinded to physician and patient identities, full blinding could not be ensured and may have introduced bias, especially as the raw audio was not modified to redact personal identifiable information.

The study did not include a systematic assessment of medical complexity between Arabic and English encounters, which may influence the interpretation of between-group comparisons. Future studies should capture encounter-level characteristics, such as number of medical entities spoken and number of diagnoses, to assess whether any observed language–based differences may be confounded by case complexity. Additionally, encounters were classified as Arabic or English based on the predominant language, without quantifying the degree of code-switching within individual consultations. This may limit the understanding of the model’s performance in heavily code-switched versus predominantly monolingual encounters. Separately, a formal safety audit evaluating the potential clinical impact of documentation inaccuracies was not performed. Incorporating such assessments in future work may further strengthen the evidence base. While the training data included multiple Arabic dialects, performance was not systematically evaluated across specific dialect groups, limiting conclusions regarding dialect-level generalizability.

The physician experience survey was based on a convenience sample of clinicians who had used Sahl AI in at least 3 clinical encounters, which may introduce selection bias toward more engaged users. As the survey was distributed through direct outreach during deployment and the total number of physicians invited was not systematically recorded, a formal response rate could not be calculated. Consequently, the survey findings should be interpreted as descriptive user feedback rather than generalizable estimates of physician satisfaction or adoption. As such, the specific percentages reported from the survey should not be interpreted as representative of the broader physician population.

Since the completion of this study, subsequent iterations of the Sahl AI models have been released with improvements in accuracy and processing speed. Further research is needed to evaluate the clinical impact of Arabic medical ambient AI scribes, including objective measures of time savings, documentation quality compared with standard practice, effects on physician-patient interaction, and downstream operational and revenue-cycle outcomes.

### Conclusions

This study highlights the potential of medical ambient AI scribe technology to enhance clinical documentation in both Arabic and English clinical environments. Sahl AI demonstrated the ability to generate high-quality clinical notes, with potential benefits including improved documentation quality, time savings, and reduced cognitive burden for physicians. To support broader adoption, future research should address the limitations identified in this study, including evaluation across diverse clinical settings and patient populations, as well as integration with electronic medical record systems. Importantly, this work provides a foundational framework for the development and training of medical ambient AI scribes in low-resource languages, such as Arabic, while also introducing a robust methodology for assessing the quality of AI-generated clinical documentation.

## Abbreviations

AI: artificial intelligence
EMR: electronic medical record
ICC: intraclass correlation coefficient
MAD: mean absolute difference
PDPL: Personal Data Privacy Law
PDQI-9: 9-item Physician Documentation Quality Instrument
